# Evaluation of prognostic utility of MIB-1 and p53 expression in pituitary adenomas: correlations with clinical behaviour and follow-up results

**DOI:** 10.1080/13102818.2014.932510

**Published:** 2014-08-22

**Authors:** Asen Hadzhiyanev, Radina Ivanova, Emil Nachev, Atanaska Elenkova, Maria Yaneva, Sabina Zaharieva, Marin Marinov, Jivko Surchev, Asya Ivanova

**Affiliations:** ^a^ Department of Neurosurgery, University Hospital ‘St. Ivan Rilsky’, Medical University of Sofia, Sofia, Bulgaria; ^b^ Clinical Center of Endocrinology and Gerontology, Medical University of Sofia, Sofia, Bulgaria; ^c^ Medical University of Sofia, Sofia, Bulgaria

**Keywords:** pituitary adenoma, immunohistochemistry, proliferation rate, p-53 expression

## Abstract

Pituitary adenomas (PAs) show a broad clinicomorphological spectrum. The proliferation activity, evaluated by MIB-1 labelling index (LI), and p53 expression have been pointed as predictive markers for invasiveness and progression. The aim of this study was to evaluate the proliferation rate and p53 expression and to look for any relationships with the clinical behaviour and follow-up results in a series of Bulgarian patients with PAs. A total of 93 patients with PAs (81 hormone-secreting, 12 non-functioning), who were operated on and followed up for a period of five years, were included. The MIB-1 LI and p53 expressions were determined by immunohistochemistry and correlated with various clinical and tumour variables. The whole group of PAs showed a low proliferation rate with evident variations in a small number of cases (MIB-1 LI – 0.50 ± 0.56, from 0.1 to 3.30). MIB-1 LI correlated with tumour size (*p* = 0.012) and was positively related with male gender (*p* = 0.23) and partial surgical resection (*p* = 0.036). We found no significant differences regarding the age, functional activity, invasion (*n* = 33), expansion (*n* = 37) and tumour recurrences (seven cases). Only 10 cases (10.8%) showed a focal, nuclear p53 immunoreactivity. The p53 positive tumours had higher proliferation rate (*p* = 0.0001) but no relationship with the other clinical and tumour variables. Among all cases, there was only one case with higher MIB-1 LI (3.3%), positive p53 expression and tumour recurrence after surgery. Our results show that most PAs have a low proliferation rate and lack of p53 expression, as well as no relationship with tumour invasion or postsurgical progression.

## Introduction

Pituitary adenomas (PAs) are common neurosurgical lesions and have been reported to account for 10%–15% of all brain tumours.[1] They are most commonly encountered in patients between the third and fourth decade of life and can be present in both genders. Their clinical presentation is connected to the abnormal secretion of hormones (functioning adenomas) and/or the mass effect they have on neighbouring neural structures. The lesions that are big enough to have a clinically significant mass effect are most commonly non-functioning. PAs are highly differentiated tumours and arise from a certain cell type of anterior pituitary. Depending on the originating cell, the PA can secrete growth hormone (GH), adrenocorticotrophic hormone (ACTH), prolactin (PRL), thyroid-stimulating hormone (TSH) and gonadotropins – luteinizing hormone (LH) and follicle-stimulating hormone (FSH). The signs and symptoms of the particular disease that a functioning PA causes are the result of the specific hormonal abnormalities. Surgical treatment is complex and multidisciplinary. It consists of a thorough preoperative workup (physical examination, hormone panels and standard preoperative laboratory panels), surgical resection and postoperative follow-up of physical condition and hormone levels. Appropriate treatment with medication is also considered.

PAs show a broad clinicomorphological spectrum – from microadenomas to tumours with local invasion and recurrence after surgery. Tumour size, invasion and the adequacy of resection have been considered as important risk factors for recurrence or progression.[2] The growth of the pituitary tumour depends on the balance between the proliferating cells and the loss of tumour cells by apoptosis and ischemic or haemorrhagic events. The determination of cell proliferation activity has been suggested to be useful in making prognosis in anterior PAs. Landolt et al. [3] were the first who determine the proliferation-associated antigen Ki 67 in fresh-frozen specimens in a series of PAs. Ki-67 is a nuclear antigen expressed in G1, G2 and synthesis phases of the cell cycle but not in the resting G0 phase.[4] Today, the MIB-1 monoclonal antibody is the most commonly used one for the purpose of immunocytochemical Ki 67 staining and evaluation in routinely processed paraffin-embedded tissue specimens of PAs. The last World Health Organization Tumour Classification of Tumours of Endocrine Organs defines an atypical PA as a tumour with a Ki-67 labelling index (LI) higher than 3% and extensive p53 positivity.[5] But the actual value of these markers in daily practice is controversial. One reason for this is that tumours with an LI higher than 3% and extensive p53 expression are uncommon, even in tumours with postsurgical progression. Conversely, their absence does not exclude clinically relevant aggressive behaviour.[6] There is no clear relationship between immunohistochemical p53 overexpression and progression of PAs.[7,8] Abnormally, high levels of expression of p53, a tumour suppressor protein that in humans is encoded by the *TP53* gene, are found in many cancers. It has been reported that almost all pituitary carcinomas express p53 whereas only 5%–25% of invasive PAs express p53.[9]

The aim of this study was to evaluate the proliferation activity and p53 expression in a large series of Bulgarian patients with surgically treated PAs. The relations between proliferation activity and p53 expression and tumour characteristics and progression were also investigated.

## Materials and methods

### Patients’ characteristics

A total of 93 patients with surgically resected PAs and followed-up for a period of five years were included. Evaluation of the MIB-1 LI and p53 expression of surgical specimens was performed by immunohistochemistry in all cases.

The mean age of the patients was 42.6 ± 13.09 years (ranged from 16 to 73 years). They were 33 males and 60 females (M:F ratio 1:1.81). According to endocrine assessment, there were 81 patients with functioning adenoma and 12 with non-functioning adenomas. Among the functioning PAs, there were 47 (58%) GH-, 20 (25%) PRL- and 14 (17%) ACTH- secreting tumours. The neuroradiological diagnosis was obtained using contrast-enhanced cerebral CT scan and MRI. A transsphenoidal surgical approach was used in all cases. The mean size of PAs was 21.0 ± 16.9 mm. According to the tumour size, the tumours were classified as microadenoma (<10 mm in diameter) – 34 cases (36.6%); mesoadenoma (between 10 and 20 mm in diameter) – 27 cases (29.0%) and macroadenoma (>20 mm in diameter) – 32 cases (34.4%). Tumour expansion above the *sella turcica* was recorded in 56 out of the 93 cases (60.2%). A surgically verified tumour invasion of *dura* was identified in 60 out of the 93 cases (64.5%). A total surgical removal of the tumour was done in 67 cases (72.0%) and partial incision in the remaining 25 cases (28.0%). The histological examination showed characteristic features of an anterior PA in all cases. All patients were subjected to close clinical and hormonal postoperative surveillance. The recurrence rate for the follow-up period was 7.5% (in 7 out of the 93 cases). Recurrence of endocrine pathology was evident in four cases with functioning adenomas, all with partial resection. Recurrence of mass-effect-related symptoms was present in 3 out of the 12 patients with non-functioning adenomas.

### Immunohistochemistry

Immunohistochemical staining for MIB-1 and p53 was done on serial 5-μm sections from paraffin-embedded tumour tissue by avidin–biotin–streptavidin method using monoclonal MIB-1 and p53 antibodies (Biogenex, USA). MIB-1 LI has been calculated as the percentage of MIB-1 positive cells (brown staining of the nuclei) per 1000 tumour cells. The counting of cells was done manually on two high-power fields (400*x*) with the highest number of MIB-1 positive nuclei (hot spots). The expression of p53 was graded semi-quantitatively in three categories: severe (when most of the cells are positive), moderate (when approximately 50% of the cells are positive) and weak/focal (when scattered positive cells are visible).

The MIB-1 LI and p53 expression were correlated with various clinical and tumour variables: age, sex, tumour size, tumour expansion, tumour invasion, volume of surgical resection and recurrence.

### Statistical analysis

All data were analysed with SPSS software using descriptive, Student's *t*-test, ANOVA, Mann–Whitney and correlation tests. A *p*-value less than 0.05 was considered statistically significant.

## Results and discussion

**Figure 1.**
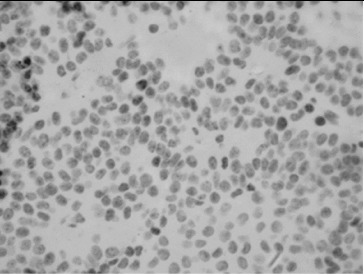
Single MIB-1 positive nuclei in a case with prolactinoma (IHC, x100).

**Figure 2.**
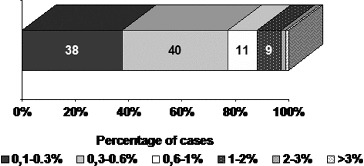
Distribution of MIB-1 LI among the cases with pituitary adenomas.

All PAs showed single MIB-1 positive nuclei, with some variations in small number of cases (Figure 1). The mean value of MIB-1 LI for the whole group of PAs was 0.50 ± 0.56, and ranged from 0.1 to 3.30. The value of MIB-1 LI was in 80 cases less than 1%, in 2 cases (2.2%) – above 2%, and only in 1 case (1.1%) – above 3% (Figure 2).

**Figure 3.**
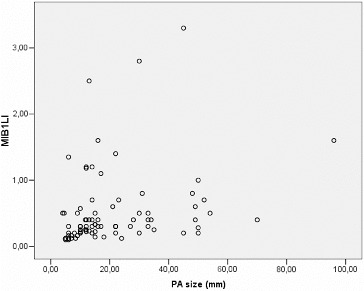
MIB-1 LI according to the size of pituitary adenoma.

**Figure 4.**
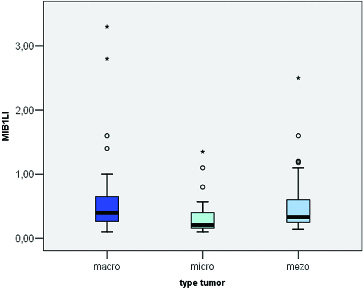
Mean values of MIB-1 LI according to the type of pituitary adenoma.

**Figure 5.**
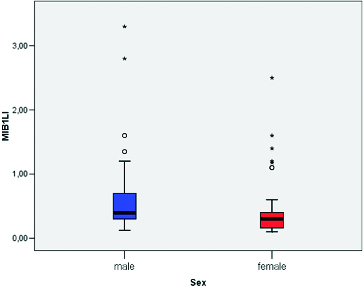
Mean values of MIB-1 LI according to the sex of patients with pituitary adenoma.

**Figure 6.**
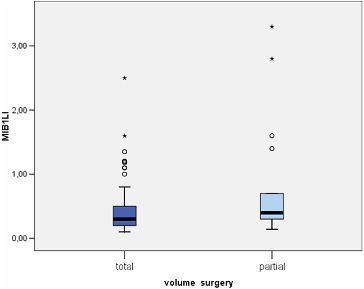
Mean values of MIB-1 LI according to the volume of surgical removal of pituitary adenoma.

MIB-1 LI correlated positively with the size of the tumour (Pearson correlation coefficient 0.275, *p* = 0.012) (Figure 3). We found a significant difference between the mean values of MIB-1 LI of microadenomas and mesoadenomas (*p* = 0.007) and macroadenomas (*p* = 0.008) (Table 1, Figure 4). MIB-1 LI was higher in male subjects compared to female ones (*p* = 0.23) (Figure 5). There also was a significant difference of MIB-1 LI in relation to the volume of surgical resection (*p* = 0.036) (Figure 6). However, the differences of the index in relation to age, tumour functional activity, expansion or invasion, and also presence of recurrence were not significant (Table 1).

**Table 1.** Relationships between MIB-1 LI and clinical behaviour of PA.

Parameters	MIB-1 LI (*x* ± S)	*p* = (ANOVA)
Tumor size		
Microadenoma	0.32 ± 0.28	
Mesoadenoma	0.57 ± 0.55	**0.007**
Macroadenoma	0.63 ± 0.73	**0.008**
Sex		
Male	0.68 ± 0.7	
Female	0.40 ± 0.43	**0.023**
Surgery volume		
Total	0.44 ± 0.43	
Partial	0.74 ± 0.85	**0.036**
Functional activity		
No	0.61 ± 0.39	0.488
Yes	0.49 ± 0.59	
Tumor expansion		
No	0.41 ± 0.44	
Yes	0.65 ± 0.69	0.061
Tumor invasion		
No	0.45 ± 0.43	
Yes	0.62 ± 0.74	0.170
Postsurgical recidive		
No	0.51 ± 0.58	
Yes	0.58 ± 0.43	0.717

**Figure 7.**
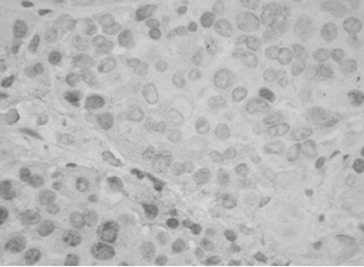
A focal p53 positive reaction in a case with non-functioning PA (IHC, x100).

Regarding the p53 expression of PA, only 10 cases (10.8%) showed focal, nuclear p53 immunoreactivity (Figure 7). The p53 positive tumours had higher proliferation rate (1.19 ± 0.9, *p* = 0.0001). However, there was no significant difference of p53 expression in relation to clinical and biological variables (data not shown). Among all cases, there was only one case with high MIB-1 LI (3.3%), positive p53 expression and tumour recurrence after surgery.

Many researchers in last years have mainly studied the MIB-1 LI of PAs and its relationship with invasiveness and recurrence of the tumour. Although comparisons of the results of different series are difficult since the variations of studied patients, there is no clear answer on the clinical usefulness of immunohistochemical measurement of the proliferation rate in PAs. For this reason we evaluated the potential prognostic value of MIB-1 LI and p53 expression in a series of Bulgarian patients with PAs who were surgically treated and followed up for a mean period of five years.

Our results confirmed the previous data that most of the PAs have very low proliferation rate. Other authors also reported mean Ki-67 LI to be approximately 1% and the highest LIs to be 2.8%, 3.7% and 4.6%.[3,10,11] In series of Pas, Zhao et al. [12] and Mastronardi et al. [13] have shown the mean MIB-1 LI to be 1.4% and 2.64%, respectively. Also, it has to be mentioned that MIB-1 LI depends on tissue processing, staining method, assessment method (digital image analysis or manual cell counting), and is also subject to interobserver variability.[14,15]

The current study also attempted to look for any relation between the MIB-1 LI and various demographic, clinical and tumour variables of the patients with PAs. A correlation between MIB-1 LI and patient age was not found. The mean MIB-LI in men was significantly higher compared to women. The literature data regarding the relationship between the growth rate and age, and sex are inconsistent. Some authors report a significantly high MIB-1 LI in patients below 30 years of age and also an inverse correlation between patient age and MIB-1 LI.[16] The opposite was found in a study that described high Ki-67 LIs in elderly patients with non-functioning adenomas.[17] Another study, however, compared different age groups (<25 years, 25–50 years and >50 years), and did not establish significant difference in the Ki-67 LI.[18] Our results regarding the gender are similar to those of Delgrange et al.[19] They observed that in prolactinomas, proliferation indices were slightly higher in males than in females but not statistically significant. Some other authors report that MIB-1 LI is not related to patient gender.[3,15,20]

A positive correlation between the MIB-1 LI and tumour size was found by us in the present investigation. The mean levels of MIB-1 LI of mesoadenomas and macroadenomas were higher compared to that of microadenomas. MIB-1 LI was also higher among the tumours with in comparison to tumours without expansion, but the difference did not reach statistical significance. The relationship between tumour size and tumour cell proliferation described so far in literature is unclear. One study also reported a higher proliferative activity in macroadenomas.[2] However, other studies failed to demonstrate such relationship.[3,19] Mastronardi et al. [21] reported similar values of mean MIB-1 LI for microadenomas, intrasellar macroadenomas and intra-suprasellar macroadenomas. The authors found that the mean LI of intra-supra-parasellar macroadenomas was higher compared to other subgroups but probably reflects their higher incidence of invasiveness. They concluded that, with the exception of invasive adenomas, the different tumour size of PA seemed to be correlated to the time of growth rather than to a different growth fraction.

According to our results, the mean level of MIB-LI was higher in non-functioning tumours compared to functioning PA, but there was not a statistical significance. Again there is no agreement in published studies.[22] While some investigators did not detect any difference in Ki-67 between hormonally active and inactive tumours, others reported higher values for active tumours.[15]

PAs frequently invade surrounding structures although they are not considered malignant. The reported frequency of invasive PAs has greatly varied from 10% to 85%, a reflection of different criteria for the definition of invasiveness. In our series, tumour invasion during surgery was identified in more than half of the patients (65%). The mean MIB-1 LI was higher in invasive tumours compared to non-invasive ones but the difference was not significant. Previous publications have also shown that PAs with higher Ki-67 LIs are more often presenting with invasion of *dura mater*.[11,15] Other studies that evaluate the proliferation rate of PAs have made an effort to define an index value that could serve as a cut-off in order to distinguish between invasive adenomas of benign behaviour and those of aggressive behaviour. Thapar et al. [15] reported that MIB-1 LI of 3% was successful in distinguishing between invasive and non-invasive adenomas with a specificity of 97% and a sensitivity of 73%. Mastronardi et al. [13] established two different thresholds for the Ki-67 index: one for invasive adenomas (3.5%) and the other for adenomas with invasion of the cavernous sinus (5%). The analysis of the current series showed that it was not possible to establish a value that distinguishes invasive from non-invasive adenomas because of the considerable overlap of MIB-1 values between these two groups.

According to the data from the study, we observed a relation of MIB-1 LI with the extent of surgical removal. The mean MIB-1 LI was significantly higher in patients with partial excision of the tumour compared to those with total removal. It is well known that residual tumour tissue frequently shows regrowth potential. During the follow-up period of this study, recurrence of clinical symptoms after surgery was present in seven patients, all with partial tumour resection. Although their MIB-1 LI was higher compared to those with non-progressive residual tumour, the difference was without statistical relevance. Other authors also failed to demonstrate a relationship between proliferation rate and recurrence of PAs.[23] On the other hand, Abe et al. [24] reported significantly more often tumour regrowth in patients with a Ki-67 LI higher than 1.5%. Consequently, a high Ki-67 LI might suggest the need for more frequent clinical and neuroimaging follow-up, and may guide other aspects of the overall therapeutic strategy.

Expression of p53 in our study was observed in small number of cases. There was no relation of p53 expression with all other clinical and tumour parameters, except with the proliferation rate. Our results confirmed the data from previous studies for the low sensitivity of p53 in predicting biologic behaviour of PAs and the need of future studies of large series of PAs to evaluate its real prognostic value.[7–9]

## Conclusions

Most of the PAs that are included in our series have a low proliferation rate as assessed by MIB-1 LI and lack of p53 overexpression. We did not find a relationship between the tumour proliferation rate and its invasion, expansion or postsurgical progression.

In spite of all, in cases with residual tumour after surgery, immunohistochemical investigation of MIB-1 LI may be useful to indicate tumour growth and aggressiveness and to be in favour of therapeutic decision-making.
